# Bioterrorism-related Inhalational Anthrax in an Elderly Woman, Connecticut, 2001

**DOI:** 10.3201/eid0906.020728

**Published:** 2003-06

**Authors:** Kevin S. Griffith, Paul Mead, Gregory L. Armstrong, John Painter, Katherine A. Kelley, Alex R. Hoffmaster, Donald Mayo, Diane Barden, Renee Ridzon, Umesh Parashar, Eyasu Habtu Teshale, Jen Williams, Stephanie Noviello, Joseph F. Perz, Eric E. Mast, David L. Swerdlow, James L. Hadler

**Affiliations:** * Centers for Disease Control and Prevention, Atlanta, Georgia, USA; † Connecticut Department of Public Health, Hartford, Connecticut, USA; ‡ New York State Department of Health, Albany, New York, USA

**Keywords:** *Bacillus anthracis*, inhalational anthrax, bioterrorism, postal facilities, research

## Abstract

On November 20, 2001, inhalational anthrax was confirmed in an elderly woman from rural Connecticut. To determine her exposure source, we conducted an extensive epidemiologic, environmental, and laboratory investigation. Molecular subtyping showed that her isolate was indistinguishable from isolates associated with intentionally contaminated letters. No samples from her home or community yielded *Bacillus anthracis*, and she received no first-class letters from facilities known to have processed intentionally contaminated letters. Environmental sampling in the regional Connecticut postal facility yielded *B. anthracis* spores from 4 (31%) of 13 sorting machines. One extensively contaminated machine primarily processes bulk mail. A second machine that does final sorting of bulk mail for her zip code yielded *B. anthracis* on the column of bins for her carrier route*.* The evidence suggests she was exposed through a cross-contaminated bulk mail letter. Such cross-contamination of letters and postal facilities has implications for managing the response to future *B. anthracis–*contaminated mailings.

On November 19, 2001, a suspected case of inhalational anthrax in a 94-year-old woman was reported to the Connecticut Department of Public Health (CTDPH) (*1*–*3*). This was the first case of *Bacilus anthracis* infection reported to the CTDPH since 1968 and the eleventh inhalational anthrax case in the United States since October 4, 2001 (*1*–*6*). The patient’s symptoms of fever, fatigue, malaise, dry cough, and shortness of breath began 20 days after the last confirmed inhalational anthrax patient became ill and 36 days after the last known intentionally contaminated letters, addressed to U.S. Senators Thomas Daschle and Patrick Leahy, were postmarked in Trenton, New Jersey (*1*–*4*) (Figure 1).The patient in Connecticut was not in the known categories of intentionally contaminated letter recipients and was not a postal worker or a mailhandler (*1*,*5*). This report describes the epidemiologic and environmental investigation conducted to determine whether her case was related to the other bioterrorism-related cases; whether she was the only case in Connecticut or a sentinel of a larger outbreak; and the source, place, and time of her exposure. The clinical aspects of the case have been described (*2*,*3*).

**Figure 1:**
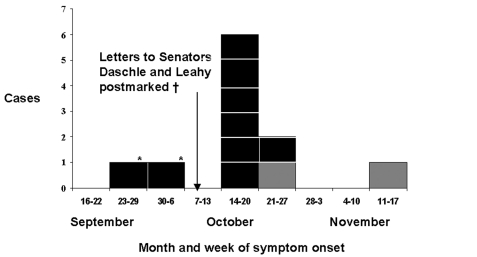
Bioterrorism-related inhalational anthrax cases by week of symptom onset—United States, 2001.The first two cases of inhalational anthrax occurred in Florida. Though no direct exposure source was found, environmental samples of the media company in which these two patients worked and the postal facilities serving the media company yielded *Bacillus anthracis* spores specifically implicating a *B. anthracis*–containing letter or package (*4*): †, the letters to Senators Thomas Daschle and Patraick Leahy were postmarked in the Trenton, New Jersey, processing and distribution center on October 9, 2001; black bars indicate cases of inhalational anthrax in persons with direct exposure to a *B. anthracis*–containing letter; gray bars indicate cases of inhalational anthrax persons with no known *B. anthracis* exposure.

## Methods

### Isolate Comparison

A subculture of the patient’s *B. anthracis* blood culture isolate was examined for species confirmation, antibiotic susceptibility testing, and molecular subtyping by multiple-locus variable-number tandem repeat analysis, which examines eight loci on the *B. anthracis* genome (*7*,*8*). The isolate was compared with previous bioterrorism-related isolates on the basis of antibiotic susceptibilities and molecular subtyping.

### Surveillance

We conducted retrospective surveillance for additional cases of human or animal anthrax in Connecticut for September 1 to November 30, 2001, by using data from death certificates; medical examiner, laboratory, and postal worker absentee records; and surveys of licensed veterinarians. We conducted prospective surveillance for additional cases of human or animal anthrax in Connecticut from November 20 to December 21, 2001, by using reports from hospital admissions, laboratories, healthcare providers, veterinarians, and animal control officers, and also reports from the U.S. Postal Service (USPS) on employee absenteeism (*9*,*10*).

### Patient Epidemiologic Investigation

In collaboration with local, state, and federal law enforcement agencies, we identified the patient’s activities, home visitors, and all places she visited in the 60 days preceding her symptom onset using her personal calendar and interviewing her family, friends, neighbors, physicians, and persons who cleaned her home. We also met with investigators of the 10th inhalational anthrax case from New York City to assess similarities between the two cases.

### Patient Environmental Investigation

In the patient’s home, environmental samples and selected personal effects were collected for culture during eight inspections conducted from November 20 to December 4. We obtained swab and wipe samples from clean, nonporous surfaces and vacuum samples from large or dusty nonporous or porous surfaces (*11*). Surface swab samples were collected by using synthetic swabs moistened with sterile saline or sterile water to sample such surfaces as vents; furniture; appliances, including vacuum cleaners; areas with dust; electrostatically charged surfaces, including a television screen; aerosolizing and misting devices, including an inhaler and a perfume bottle; and all places in the home where she might have handled her mail. Vacuum samples were collected by using high-efficiency particulate air (HEPA) vacuum cleaners equipped with a filter collection device to vacuum carpets, furniture, and clothing. Final intensified sampling was performed by using blowers to aerosolize particles throughout the living space, followed by air sampling that used high-volume air filtration devices and the placement of blood agar settle plates throughout the home. Personal effects collected from the home included pieces of mail, file folders, pieces of paper, used tissues, letter openers, pill bottles, an inhaler, photographs, and a calendar. Bulk samples of contents from the bags of vacuum cleaners normally used to clean the home were collected for culture. Nasal swabs were taken from all persons who spent >60 minutes in the patient’s home during the 60 days before onset of her symptoms.

Outside the home, environmental samples were collected for culture from November 20 to December 2. We collected moist swab and vacuum samples from all indoor air spaces she had visited in the 60 days preceding her symptom onset. Moist swab samples were also collected at selected outdoor locations, including her mailbox and the soil around it, mailboxes on her street, soil in her yard, and soil at a local establishment rumored to be located on the site of a previous farm that was closed because of an anthrax outbreak among cattle during the early 1900s.

### Postal Epidemiologic Investigation

USPS has a mail tracing system by which data are recorded from bar codes applied to letters in postal processing and distribution centers (PDCs). For first-class letters, canceling machines apply both an identification code (orange bar code on the back of the envelope) and a postnet code (black bar code on the front of the envelope) to envelopes. Bulk letters are not processed on canceling machines because they are presorted with the postnet code preapplied. When the identification code is used, canceling machines record the day of the month, the time of day, and the sequence in which each first-class letter was processed during a given half-hour interval. Sorting machines then use the postnet code to group both first-class and bulk letters that go to a particular address. Sorting machines record the total count of all letters processed on each sorting machine, but only first-class letters with identification codes include time of day and identity of the particular sorting machine. Consequently, identifying information on bulk letters is not recorded in the mail tracing system. All letters are processed on sorting machines approximately two to five times, progressing from an initial sort, to the five-digit zip code, through a specific final sort in which letters are sorted to collecting bins in the delivery sequence for the given postal carrier route (*12*)*.*

We searched the patient’s home for letters she received since September 1, 2001, and recovered letters from her office, personal files, and trash bins. The recovered letters were categorized as first-class or bulk and submitted to the CTDPH laboratory for culture. First-class letters were checked against the USPS database for date and location of cancelation. To determine whether the patient received first-class letters from any PDCs that processed an intentionally contaminated letter (i.e., Trenton, NJ; Brentwood, Washington, DC; Morgan, New York City; and West Palm Beach, FL), we examined USPS data of outgoing mail from these facilities for first-class letters with Connecticut destination addresses. Data were reviewed from October 9 until either the date the particular PDC closed because of *B. anthracis* contamination or, if the PDC did not close, the date the patient’s symptoms began (November 13). We chose October 9 as a starting date because the only recovered intentionally contaminated letters that resulted in cases of inhalational anthrax were postmarked on this date and because 36 days had already elapsed between this date and the onset of the patient’s symptoms. Information on the date, time, and machines involved in processing these first-class letters was retrieved.

To identify all first-class letters the patient received, regardless of point of origin, we examined USPS data for first-class letters sorted in the Southern Connecticut PDC that served her local post office from October 9 to November 13. We retrieved available information on the date, time, and identity of a letter’s originating PDC; however, information on the machines that processed a particular letter in the originating PDC is not available. To check for bulk letters sent to the patient’s zip code from October 9 to October 16, 2001, we contacted mailing companies that used the Trenton PDC. To check for bulk letters sent to the patient’s zip code during October 9 to October 16, 2001, we contacted mailing companies that used the Trenton PDC.

### Postal Environmental Investigation

Environmental samples were collected at the Southern Connecticut PDC on five occasions. On November 11, 2001, 2 days before the patient’s symptoms began, an independent contractor took surface samples from various locations with dry synthetic swabs as part of a nationwide USPS effort to identify contamination of selected PDCs. On November 21, a second independent contractor hired by USPS collected additional dry swab samples of surfaces, including 29 letter-canceling and -sorting machines, 4 flat- (magazine) sorting, and 4 parcel-sorting machines; air-handling units; and vacuum cleaner filters from different facilities. On November 25, we inspected and repeated sampling of similar locations using moist synthetic swabs. On November 28, guided by findings from epidemiologic investigations at the Brentwood PDC, we collected vacuum and moist synthetic 2x2-inch surface wipe samples from all letter-canceling and -sorting machines (*13*). After samples from three letter-sorting machines were positive for *B. anthracis,* we collected additional moist surface wipe samples from each column of collecting bins on the three letter-sorting machines with samples yielding *B. anthracis* and from the sorting machine that completed the final sort of letters that included the patient’s mail carrier route. We also collected nasal swabs for culture from employees of the Southern Connecticut PDC during November 21 to 24.

At the patient’s local post office, we obtained dry and moist synthetic surface swab and vacuum samples of the mail-sorting area, computer screens, gurneys, carts, loading dock, and vehicle serving her postal carrier route on four occasions during November 21 to December 2, 2001. We collected nasal swabs for culture from employees of her local post office during November 21 to November 24.

### Postal Laboratory Studies

All environmental samples were tested either in the CTDPH laboratory or, when it had reached its capacity, a contract laboratory in Texas. Surface swab, nasal swab, and vacuum samples and blood agar settle plates were analyzed in the CTDPH laboratory. Surface wipe, vacuum, and air-filter samples, and bulk contents of vacuum cleaners were analyzed in the contract laboratory in Texas. All swab specimens were plated directly onto sheep blood agar and handled using standard procedures (*14*). Vacuum, air-filter, and surface wipe samples, as well as vacuum cleaner bag contents, were processed with the following extraction procedure: the specimen contents were placed into a sample-processing solution and centrifuged to create a pellet; the pellet was resuspended in 0.3% Tween 20 in phosphate-buffered saline; the resuspended solution was then heat-shocked; and one tenth of the resuspended solution was placed on sheep blood plates (*14*). All suspicious colonies were screened by Gram stain and motility testing and confirmed by gamma phage lysis and polymerase chain reaction (*14*). All samples from the patient’s home that underwent the extraction procedure and tested negative were retested with one half of the remaining sample-processing solution.

## Results

### Isolate Comparison and Surveillance

Molecular subtyping results identified the isolate as being of MLVA genotype 62, and antibiotic susceptibilities of the isolate were indistinguishable from those of the other human anthrax patients confirmed nationwide since October 4, 2001 (*4*,*8*). Retrospective and prospective surveillance did not identify any additional human or animal anthrax-related illness (*9*,*10*).

### Patient Epidemiologic Investigation

The patient lived in a central Connecticut town with a population of 9,821 persons (*2*). She lived alone in a ranch-style home with a basement, located on a half-acre, partially wooded lot on a residential side street approximately one third of a mile from the state road. The home entryway was readily visible to homes across the street.

She spent most of her time inside her home reading or watching television and did not spend time outdoors. She did not shop or cook; family and friends provided food and household supplies. Her home was clean and well-organized. She had no hobbies requiring woolen items (e.g., knitting or crocheting), goat hair, or leather. A family member assisted her in paying bills, reading unfamiliar letters, and handling bank transactions. In the 60 days preceding symptom onset, her only visitors besides family and close friends were her church pastor, approximately 25 Halloween trick-or-treaters, and two persons from her cleaning service. No unusual visitors or solicitors were noted by friends or neighbors.

She did not drive; family and friends provided transportation. She was always accompanied when outside the home and limited her time away from home to <2 hours to prevent fatigue. Her activities outside her home consisted of weekly hair salon appointments, routine physician visits, lunch outings with friends, weekly church attendance, visiting another church for a Christmas fair, and voting at the town hall. No unusual persons or occurrences were noted by those who accompanied her during these outings.

When she was compared with the 10th case-patient with inhalational anthrax in New York City, limited similarities were found: both were women >60 years of age, lived alone in clean homes, wore hats, and had a bottle of the same brand of perfume (which was sampled for the presence of *B. anthracis*). They had no brand of medication, physicians, hobbies, social networks, or geographic area in common.

### Patient Environmental Investigation

Cultures of all 258 samples and 84 personal effects from the patient’s home were negative for *B. anthracis.* The 181 samples from the indoor air spaces she visited in the 60 days preceding symptom onset (including 11 restaurants, seven cars, five physician’s offices, five homes of neighbors or close friends, two churches, a bank, a hair salon, and a public building) also cultured negative for *B. anthracis.* Cultures of the 17 samples from the selected outdoor locations and 16 nasal swabs from visitors to her home also were negative for *B. anthracis.*

### Postal Epidemiologic Investigation

We recovered 29 letters from her home postmarked after September 1, 2001: 7 canceled first-class letters and 23 presorted bulk letters. These 29 letters likely did not represent the entire number of letters she had received since September 1. Of the six first-class letters, only one was postmarked after October 9 (October 26). All first-class letters were sliced open along the top border of the envelope, whereas bulk letters, mainly solicitations or credit-card offers recovered from her garbage, had been torn in half. Samples from all 29 recovered letters were negative for *B. anthracis*.

The Morgan and West Palm Beach PDCs did not save records of outgoing mail for the period of interest. The Trenton and Brentwood PDCs did not send first-class letters directly to her address during October 9 to October 21 (date after which both facilities were closed because of *B. anthracis* contamination). Five first-class letters from the Trenton PDC and three first-class letters from the Brentwood PDC were sent to her postal carrier route during this time. Two of the letters from the Trenton PDC were processed approximately 3 hours after the Daschle and Leahy letters and on the same canceling machine. None of these letters were delivered to an address on her street, none coincided with a first-class letter to the patient, and none were recovered.

USPS data from the Southern Connecticut PDC showed that eight first-class letters, only one of which was recovered, had been sent to her address during October 9 to November 13, 2001. All eight originated in Connecticut and were canceled at the Southern Connecticut PDC.

While examining the USPS data, we identified and recovered a first-class letter (letter A) that was sorted in the Trenton PDC 283 letters (approximately 15 s) after the intentionally contaminated letter to Senator Leahy. Letter A was processed in the Southern Connecticut PDC and the patient’s local post office and delivered to an address approximately 4 miles away on a different mail carrier route. Three separate moist swab samples taken from the outside of letter A’s envelope yielded 1, 3, and 7 CFU of *B. anthracis,* respectively. Cultures of moist swab samples from the inside of the envelope and its contents were negative for *B. anthracis*. The sorting machine in the Southern Connecticut PDC that first processed letter A could not read the postnet code. Therefore, letter A was removed from the automated system and hand-sorted. Eight moist wipe and vacuum cleaner samples from the sorting machine in the Southern Connecticut PDC that first sorted letter A were negative for *B. anthracis* by culture as were 36 moist swab and vacuum cleaner samples from the home that received letter A, including the box that the letter was stored in, the letters stored next to letter A, the home’s mailbox, and mailboxes from homes on either side of the home.

Overall, the Trenton PDC processed approximately 5 million letters between October 9–October 16, and the Brentwood PDC processed 13 million letters between October 9 and October 21, when they were closed. Of these, approximately 1.1 million letters were processed at each facility within 24 hours after the letters to Senators Daschle and Leahy passed through, half of which were bulk letters (Table 1).

**Table 1:** Volume of letters processed after the *Bacillus anthracis–* containing letters to Senators Thomas Daschle and Patrick Leahy in the Trenton, New Jersey,and Brentwood, District of Columbia, processing and distribution centers during two intervals, October 2001

Type of letter	Trenton, NJ		Brentwood, DC	
	Oct. 9–16	Oct. 10^a^	Oct. 9–21	Oct. 10^a^
Bulk letters	~3,000,000	~500,000	~6,000,000	~500,000
First-class letters	~2,000,000	~500,000	~7,000,000	~ 600,000
First-class letters to Southern Connecticut PDC	20,451	3,645	24,181	3,836
First-class letters to patient’s local post office	39	9	66	9

^a^Oct. 10 represents the volume of letters processed during the 24-hour period after the letters to Senators Daschle and Leahy were processed.

Of 33 bulk mailing companies that sent letters through the Trenton PDC, 19 sent letters to Connecticut; 17 of these companies had no records of bulk letters sent to the address of the patient or her close contacts.

### Postal Environmental Investigation

**Figure 2:**
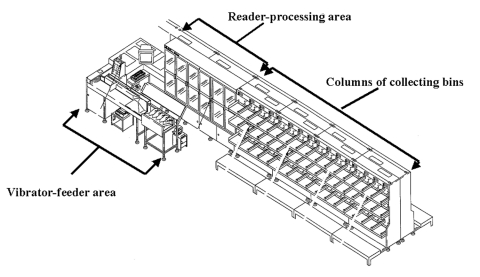
Diagram of a letter-sorting machine

The Southern Connecticut PDC is approximately 350,000 square feet. It has 11 machines to cancel and code letters originating there and 18 machines to sort letters originating in and arriving at the Southern Connecticut PDC. Each letter-sorting machine has 48–52 columns of permanent collecting bins arranged in columns four bins high (Figure 2). Flats and parcels are processed by different machines located in different areas of the PDC. The facility is highly computerized and processes approximately 3 million letters, flats, and parcels daily.

Forty-one (7%) of 590 samples from the Southern Connecticut PDC yielded *B. anthracis.* The strain was indistinguishable from that of the bioterrorism-related isolates (*9*). All of the positive samples were from 4 (31%) of the 13 final sorting machines. Sorting machine 10, normally used to sort bulk letters as they arrived at the facility, was heavily contaminated. A vacuum sample from the vibrator-feeder area yielded anthrax spores, estimated as 2.9 million *B. anthracis* CFU in 0.53 g of paper dust collected. Wipe samples of 30 (58%) of 52 columns of bins from this machine yielded *B. anthracis*. Of 65 swab, wipe, or vacuum samples collected from the sorting machine (no. 6) that completed the final sort of letters that included the patient’s mail carrier route, only the wipe sample taken from the column of bins that held sorted letters for the patient’s postal carrier route were positive for *B. anthracis* (Table 2). Cultures of the 63 environmental samples taken at the local post office and 485 nasal swabs from employees working at the Southern Connecticut PDC or the local post office were negative for *B. anthracis*.

**Table 2:** Number of positive and total samples^a^ by sampling location and date, regional processing and distribution center—Connecticut, 2001

Sampling location	Nov. 21 positive total (%)	Nov. 25 positive/total (%)	Nov. 28 positive/total (%)	Dec. 2 positive/total (%)
Letter-sorting machine 4	0/0	0/0	1/12 (8)	1/48 (2)
Letter-sorting machine 6	0/3 (0)	0/ 2 (0)	0/22 (0)	1/48 (2)
Letter-sorting machine 10	0/0	0/0	4/ 8 (50)	30/52 (58)
Letter-sorting machine 11	0/1 (0)	0/0	1/8 (13)	3/52 (6)
All letter-canceling machines (n=11)	0/6 (0)	0/4 (0)	0/99 (0)	0/0
All flats-processing machines (n=4)	0/10 (0)	0/8 (0)	0/34 (0)	0/0
All parcel-processing machines (n=4)	0/8 (0)	0/18 (0)	0/4 (0)	0/0
Other locations in regional PDC	0/10 (0)	0/2 (0)	0/4 (0)	0/0
	0/27 (0)	0/25 (0)	0/21 (0)	0/0

^a^Although multiple sampling techniques were used, exact locations were not sampled in a manner that would allow comparison of results by sampling techniques. Therefore, all types of samples are listed as a composite total. PDC, processing and distribution center.

## Discussion

This report describes the epidemiologic and environmental investigation of a case of inhalational anthrax in a 94-year-old woman from rural Connecticut. Molecular subtyping and antibiotic susceptibility testing of the isolate from blood culture demonstrated that it was indistinguishable from the other bioterrorism-related isolates, establishing a link to the cases that were caused by the mailing of intentionally contaminated letters (*3*,*7*,*8*). Surveillance efforts indicate that this was an isolated case in Connecticut and not a sentinel for a larger exposure (*9*,*10*). Although a direct exposure was not found, our investigation indicates cross-contaminated bulk mail as the source of her exposure.

Of the 11 cases of bioterrorism-related inhalational anthrax, 9 with known exposures had incubation periods of 5 to 13 days (*15*,*16*). Because 36 days had passed since the last known intentionally contaminated letters (Daschle and Leahy letters) were postmarked and the onset of the Connecticut woman’s symptoms, we considered many nonpostal potential sources of exposure: aerosolized release in her community; an intentional release at her home; and tampering of products, including pill bottles, inhalers, foods, spices, or misting devices. We also looked for similarities with the unexplained case in New York City. Extensive surveillance, epidemiologic investigation, and environmental sampling found no evidence to support these hypotheses.

We also wanted to determine whether the patient received an intentionally contaminated letter similar to the Daschle and Leahy letters, which mainly resulted in inhalational anthrax cases, rather than an intentionally contaminated letter similar to the New York City media letters, which resulted solely in cutaneous cases (*15*). Environmental sampling of the Trenton and Brentwood PDCs and the Hart Senate Office Building, which processed or received the Daschle and Leahy letters, indicated widespread contamination in these locations (*13*,*17*). In contrast, environmental sampling showed only focal contamination in the Southern Connecticut PDC and no contamination in the patient’s home, making receipt of a letter similar to the Daschle or Leahy letters unlikely. In addition, we identified no anthrax illness among postal employees at the Southern Connecticut PDC or the local post office (*1*,*4*). Although no cases of anthrax-related illness were identified in employees of the Hart Senate Office Building, early administration of postexposure chemoprophylaxis (*17*) likely prevented this.

The recovery of a cross-contaminated letter demonstrates the plausibility of cross-contaminated letters reaching their destination with demonstrable amounts of spores still on the envelope. Though *B. anthracis* was readily cultured from the outside of letter A’s envelope, we found no evidence to indicate letter A was actively shedding or transferring spores at any point in Connecticut. This demonstrates that spores can remain on or embedded in the outside surface of an envelope without measurably contaminating the environment.

The 36-day gap from the Dashle and Leahy letters to the onset of the patient’s symptoms suggests that her incubation period may have been longer than that seen with the first nine bioterrorism-related inhalational cases. In addition, the lack of any environmental evidence of *B. anthracis* spores in her home or any of the known locations she visited in the 60 days preceding illness suggests that her exposure dose was much lower than the 50% lethal dose cited in the literature (*18*). While we cannot exclude the possibility of an additional unrecognized intentional release, our findings are consistent with evidence from studies of inhalational anthrax in nonhuman primates (*19*).

We believe the patient received either a bulk letter that was directly cross-contaminated in the Trenton PDC or a bulk letter that was secondarily cross-contaminated in the Southern Connecticut PDC. Alternatively, we cannot exclude the possibility that the patient was exposed to a bulk letter secondarily cross-contaminated by one of the eight first-class letters sent to her postal route that were processed in the Trenton or Brentwood PDC after the Daschle and Leahy letters. The fact that she tore her bulk letters in half provides a possible mechanism for releasing a limited number of spores embedded in the surface of the envelope into her breathing space, a number that was too small to detect during environmental sampling of her home.

The possibility of cross-contaminated letters as a cause of anthrax illness has been postulated (*20*). Although cross-contaminated letters are potential sources of exposure and the risk of critical *B. anthracis* exposure through cross-contaminated letters is low, our investigation did not support earlier assumptions. Specifically, we found no evidence to indicate that a first generation cross-contaminated letter (letter A) was actively shedding or transferring spores at any point in Connecticut. Thus, cross-contaminated letters can have markedly different levels of cross-contamination and potential to shed spores. An individual assessment is necessary to determine the magnitude of risk.

If we assume that the case in Connecticut, and possibly the unexplained case of inhalational anthrax in New York City (*21*), resulted from exposure to cross-contaminated letters, the overall risk for inhalational anthrax from cross-contaminated letters appears very low. Approximately 2 million pieces of mail passed through the Trenton and Brentwood PDCs during the first 24 hours after the Daschle and Leahy letters contaminated these facilities, and approximately 18 million pieces passed through before the facilities were closed. This conclusion is consistent with the expectation that the majority of letters would not be heavily cross-contaminated and that only a limited number of anthrax spores might remain on an envelope by the time it reaches its destination.

If low-dose exposure occurred through the mail, numerous persons might have had such exposures. Why illness developed in the Connecticut patient and not in others is unknown, but the reasons might include her habit of tearing mail in half before disposal, her advanced age, her history of obstructive lung disease, and her use of inhaled bronchodilators (*3*). If a future mailing of *B. anthracis* spores is recognized, persons could be advised to open letters in a well-ventilated area, avoid tearing envelopes, discard envelopes after opening, and wash hands after handling envelopes (*9*).

The finding of the approximate equivalent of 3 million spores from a vacuum sample on one sorting machine underscores the importance of maintaining the revised postal facility cleaning procedures geared to minimize aerosolization of dust (*12*). Before the mailing of intentionally contaminated letters, sorting machines were routinely cleaned by using compressed air. After investigators recognized that aerosolization of spores during cleaning might have contributed to inhalational anthrax cases among postal workers, vacuum cleaners with HEPA filters replaced compressed air cleaning of sorting machines (*1*,*4*,*13*).

A number of key limitations exist to this investigation, however. First, we do not know the threshold for detecting spores in the environment. The absence of positive cultures in the home and other places sampled in her town does not exclude the presence of *B. anthracis* spores in these locations. Nonetheless, locations that processed or received intentionally contaminated letters had widespread contamination (*13*,*17*). Also, because the patient’s home was frequently vacuumed and dusted, measurable amounts of spores could have been removed during cleaning. We tried to assess this by testing contents of vacuum cleaner bags but were still unable to obtain positive cultures. Second, because we were unable to completely trace bulk letters, we do not know if she actually received any bulk letters that passed through the Trenton PDC. Third, the investigation began >1 month after anthrax spores were introduced into the postal system. Thus, certain potential environmental evidence might have disappeared by the time our investigation began.
